# Unraveling Attention-Deficit/Hyperactivity Disorder Etiology: Current Challenges and Future Directions in Treatment

**DOI:** 10.3390/neurosci6020041

**Published:** 2025-05-06

**Authors:** Abhishek Poddar, Sreelatha Gaddam, Shivakumar Sonnaila, Venkata Suryanarayana Murthy Bavaraju, Shilpi Agrawal

**Affiliations:** 1 Department of Molecular Biology, Massachusetts General Hospital, Boston, MA 02114, USA; poddar@molbio.mgh.harvard.edu; 2 Department of Genetics, Harvard Medical School, Boston, MA 02114, USA; 3 Department of Applied Chemistry, Maharaja Sayajirao University of Baroda, Vadodara 390002, India; slatha.gaddam@gmail.com; 4 Department of Chemistry and Biochemistry, University of Arkansas, Fayetteville, AR 72704, USA; sksonnai@uark.edu; 5 Department of Industrial Engineering, College of Engineering, University of Arkansas, Fayetteville, AR 72704, USA; murthy.1809@gmail.com; 6 Department of Biomedical Engineering, College of Engineering, University of Arkansas, Fayetteville, AR 72704, USA

**Keywords:** attention-deficit/hyperactivity disorder (ADHD), neurodevelopmental disorders, neurotransmitter dysregulation, pharmacological therapies, genetic and environmental interactions, gut–brain axis, non-pharmacological interventions

## Abstract

Attention-Deficit/Hyperactivity Disorder (ADHD) is a complex neurodevelopmental disorder with a multifactorial etiology involving genetic, epigenetic, and environmental factors. This review focuses on the current understanding of these contributing elements, examining how they interact to influence ADHD development. Genetic predispositions, environmental exposures, and epigenetic modifications collectively shape the risk and manifestation of the disorder. Despite advancements in research, significant challenges remain in identifying precise mechanisms and translating them into effective treatments. The variability of symptoms across individuals, influenced by factors such as age, gender, and cultural background, further complicates diagnosis and treatment. Addressing these challenges requires a deeper investigation into the underlying causes of ADHD and the development of tailored interventions. This review aims to highlight both the progress made in understanding ADHD etiology and the current gaps in treatment approaches, calling for more targeted research and personalized therapeutic strategies.

## 1. Introduction

Attention-Deficit/Hyperactivity Disorder (ADHD) is one of the most common neurodevelopmental disorders, affecting millions of children and adults worldwide [1,2]. It is characterized by persistent patterns of inattention, hyperactivity, and impulsivity that interfere with an individual’s ability to function effectively in school, work, and social settings [3,4]. While ADHD is often recognized in childhood, it is increasingly understood as a lifelong condition, with many individuals continuing to experience symptoms into adulthood [5]. ADHD can present in various ways, depending on factors such as age, gender, and cultural background, making it a complex and multifaceted disorder to diagnose and treat [6].

The significance of ADHD extends far beyond its prevalence. It impacts multiple areas of life, including academic achievement, job performance, relationships, and mental health [7,8,9,10]. Children with ADHD are at higher risk for academic difficulties, disciplinary problems, and lower self-esteem, while adults with ADHD may struggle with employment, interpersonal relationships, and emotional regulation [7,10,11]. Additionally, untreated ADHD can lead to comorbid conditions such as anxiety, depression, and substance use disorders, further complicating the lives of those affected [12,13]. The societal impact of ADHD is also substantial, with considerable costs related to healthcare, education, and loss of productivity [14,15,16,17,18,19].

Despite the growing awareness of ADHD, significant challenges remain in terms of diagnosis and treatment. ADHD is often underdiagnosed in certain populations, such as girls and minority groups, leading to disparities in access to care and treatment outcomes [20,21]. Girls, for example, are more likely to present with inattentive symptoms, which are less noticeable than hyperactivity, often leading to delayed diagnosis or misdiagnosis [22,23,24]. Similarly, ADHD in minority populations may be overlooked or attributed to other behavioral issues, resulting in fewer children and adults in these communities receiving the treatment they need [25,26,27,28,29,30].

This review follows a narrative approach, synthesizing current advancements in understanding the multifactorial etiology of ADHD. While not a systematic review, we aimed to highlight key developments and areas for future research based on recent literature. A systematic review was deemed outside the scope of this paper due to the broad range of factors covered, but we acknowledge that such a review would be a valuable next step for focused research on specific etiological aspects.

**Figure 1:**
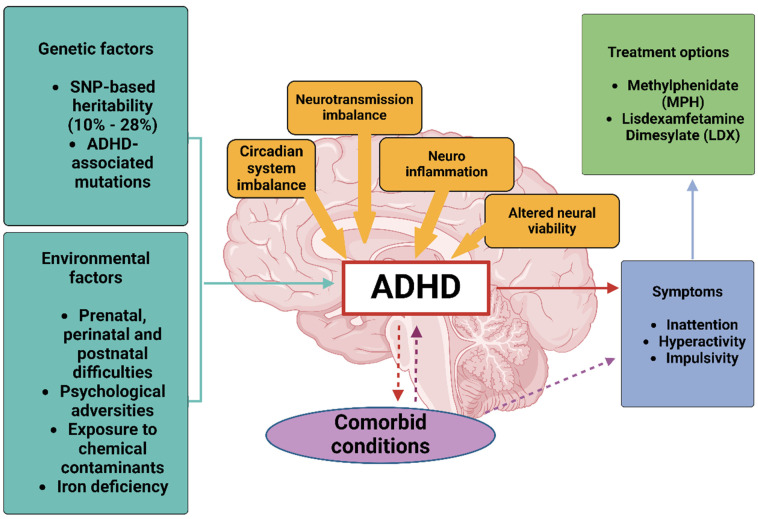
Overview of ADHD etiology and pathophysiology, highlighting genetic, environmental, and neurobiological factors influencing symptom development. This figure was created using Biorender, referencing [33].

This review aims to provide a comprehensive overview of the current understanding of ADHD, exploring the factors that have contributed to its increasing prevalence and the ongoing debates surrounding its diagnosis and treatment (Figure 1). We will also explore the wide range of treatment options available for individuals with ADHD, including both pharmacological and non-pharmacological approaches. Stimulant medications, such as methylphenidate and amphetamines, remain the most prescribed treatments [31], but alternative emerging therapies, such as cognitive behavioral therapy (CBT), behavioral interventions, emerging digital therapeutics, and the gut–brain axis, are gaining traction as complementary or standalone options [32]. By synthesizing various domains, we aim to provide a comprehensive resource for both researchers and clinicians. We conducted a comprehensive literature search of peer-reviewed articles published from 2000 to 2024, focusing on original research and review papers.

## 2. Methods

The eligibility criteria included peer-reviewed articles of any design, including narrative reviews, systematic reviews (with or without meta-analysis), original research articles, and scoping reviews. Out of approximately 856 studies screened on PubMed and 575 studies on Medline, we selected 161 (excluding duplicates, the total number of studies was 630) based on their relevance to the pathophysiology, diagnosis, genetic and environmental factors, and emerging hypotheses related to ADHD, such as the gut–brain axis. The inclusion criteria focused on peer-reviewed original research articles and review papers that addressed ADHD’s etiology or emerging research; those published in peer-reviewed journals between 2000 and 2024 were considered. We excluded non-peer-reviewed articles, studies on unrelated neurodevelopmental disorders, and papers not published in English. ChatGPT-4 has been utilized in this manuscript to assist in refining the methodology. The final content was edited to maintain accuracy and rigor.

## 3. Understanding ADHD

ADHD is characterized by a persistent pattern of inattention, hyperactivity, and impulsivity, which can significantly impact an individual’s daily functioning and quality of life [34,35,36]. The disorder typically manifests in early childhood and, if left untreated, can persist in adulthood [37]. Despite its well-documented prevalence among children and adolescents, with an estimated global prevalence of 5.29%, ADHD is often underdiagnosed, particularly in adults. Many adults with ADHD were never evaluated or diagnosed as children, and they continue to struggle with the disruptive effects of the disorder throughout their lives [37]. The symptoms of ADHD can manifest differently in adults compared to children. While hyperactivity may decrease with age, internal restlessness and difficulty with executive functions often persist. Adults with ADHD frequently struggle with time management, organization, and maintaining focus during important tasks. These challenges can affect various aspects of life, including career performance, relationships, and personal responsibilities. Many adults develop coping mechanisms to manage their symptoms, though these strategies may not always be effective.

### 3.1. Genetic Factors and Environmental Factors

In the last two decades, research into the etiology of ADHD has revealed significant progress in understanding its multifactorial causes. Both genetic and environmental contributions play critical roles, with an increasing focus on how these factors interact. The rationale for discussing both genetic and environmental factors in tandem stems from the growing body of evidence that ADHD is a complex disorder involving not just isolated genetic predispositions but also the interplay of environmental exposures that modify the genetic risk landscape. The identification of this interaction is crucial for explaining the heterogeneity in ADHD’s presentation and severity [38]. 

#### 3.1.1. Genetic Factors

ADHD has a strong heritable component, with genetic factors contributing approximately 74% to the overall risk of developing the disorder. Studies examining identical and non-identical twins have consistently shown that genetics plays a significant role in the inheritance of ADHD. However, large-scale genome-wide association studies (GWAS) have identified over 7300 genetic variants that increase risk, underscoring the polygenic nature of ADHD. Importantly, these variants alone do not guarantee the onset of ADHD, highlighting the need for an understanding of how environmental factors and gene–environment interactions contribute to the disorder’s development [38,39]. 

Key genes involved in dopamine regulation, such as DRD4 and DAT1, have emerged as central players in the genetic architecture of ADHD. These findings have been replicated across various studies, particularly through candidate gene and GWAS approaches [38,40], indicating cumulative small-risk variants that influence ADHD’s development. Candidate gene studies have identified significant polymorphisms in genes like SLC6A3 (dopamine transporter) [41], DRD4 and DRD5 (dopamine receptors) [42,43], SLC6A4 (serotonin transporter), HTR1B (serotonin receptor), and SNAP25 (synaptosome protein). Both DRD4 and SNAP25 have shown strong links to ADHD [44], each being associated in over 70% of studies, while DRD5 and SLC6A3 [43] were associated in 60% of studies. Additionally, low activity of MAOA, a gene responsible for breaking down neurotransmitters like dopamine and serotonin, amplifies sensitivity to adverse environments, contributing to a higher risk for disorders such as ADHD and conduct disorders [45,46,47,48,49].

Despite this, the overall effect size of individual variants is small, and much of the genetic risk is believed to arise from the cumulative influence of multiple small-risk variants, as indicated by polygenic risk scores (PRSs). This cumulative genetic risk suggests that genetic susceptibility interacts with environmental triggers to modulate ADHD onset, supporting the hypothesis that ADHD results from widespread dysfunction across brain networks rather than being confined to specific brain regions [50]. The LPHN3 gene, for instance, has been linked to increased susceptibility to ADHD [51], while mitochondrial DNA haplogroups have also been implicated, suggesting a role for mitochondrial mechanisms in ADHD [52,53,54,55,56]. The polygenic nature of ADHD reflects the contributions of multiple genes, each exerting small effects, but collectively influencing the risk for the disorder.

Moreover, differential susceptibility theory proposes that certain genetic variations, known as “plasticity genes”, increase an individual’s sensitivity to environmental influences, whether positive or negative. This interaction between genetic predispositions and environmental exposures is critical to understanding the complex etiology of ADHD and its comorbidities [57,58]. Understanding how both genetic and environmental factors converge can offer deeper insights into ADHD’s development and potential treatment strategies.

#### 3.1.2. Genetic Linkage Studies

Genetic linkage studies, which were among the earliest genome-wide approaches used in ADHD research, sought to identify chromosomal regions associated with ADHD inheritance within families [59]. Although early results lacked consensus on significant chromosomal regions, meta-analysis by Zhou et al. identified a region on chromosome 16, suggesting the presence of important loci related to ADHD risk [60]. These findings underscore the importance of ongoing genetic research in delineating specific risk regions, though common variants with large effects remain elusive [51].

#### 3.1.3. Candidate Gene Association Studies

Research into neurotransmission-related genes has provided additional insights into ADHD’s etiology. Dopaminergic and serotonergic pathways, both of which are targeted by current ADHD treatments, have been implicated in the disorder [61]. Meta-analyses of candidate gene studies have identified significant associations with genes such as SLC6A3 (dopamine transporter), DRD4 (dopamine receptor), and SNAP25 (synaptosome protein) [62]. A gene called BAIAP2, involved in neuronal development, was notably associated with adult ADHD [40]. These findings not only support the role of these pathways in ADHD’s neurobiology but also highlight the complexity of its genetic underpinnings.

#### 3.1.4. Genome-Wide Association Studies (GWAS)

GWAS have transformed ADHD research by allowing the identification of common variants with small but significant effects on ADHD risk [63]. The discovery of loci such as FOXP2, a gene linked to both ADHD and speech disorders, has broadened our understanding of how ADHD shares genetic pathways with other neurodevelopmental disorders [64]. The polygenic nature of ADHD is evident from GWAS findings, as multiple genes, including those involved in dopamine regulation (DUSP6) and neuronal wiring (SEMA6D), contribute to the overall genetic susceptibility [65].

#### 3.1.5. Polygenic Risk and Cross-Disorder Associations

ADHD’s genetic overlap with other psychiatric disorders, such as autism and schizophrenia, suggests shared biological mechanisms across these conditions [66]. This overlap further supports the view that ADHD exists on a continuum of neurodevelopmental variability. Cross-disorder genetic studies have revealed associations with traits such as obesity and IQ, reinforcing the idea that ADHD’s etiology involves a broad spectrum of cognitive and behavioral outcomes [67].

#### 3.1.6. The Search for Rare Genetic Variants in ADHD

While common genetic variants contribute significantly to the ADHD risk [68], research into rare genetic variants and copy number variants (CNVs) [69] has provided additional insights into the disorder’s genetic complexity [70,71]. These variants often affect pathways involved in neurotransmission, ion channel activity, and neuronal development and have been implicated in both ADHD and other neurodevelopmental disorders [72,73,74]. For example, duplications in the CHRNA7 gene, which modulates dopaminergic neurons, highlight the importance of glutamatergic and cholinergic pathways in ADHD’s pathophysiology [71,72,73,74].

#### 3.1.7. Environmental Factors and Gene-Environment Interactions

In addition to genetic influences, environmental factors such as prenatal exposure to toxins, psychosocial adversity, and diet contribute to ADHD risk [38,75,76,77]. Environmental exposures, particularly during critical developmental periods, can exacerbate or mitigate genetic susceptibility, underscoring the importance of considering both genetic and environmental contributions in understanding ADHD’s etiology [78,79,80,81,82]. Non-chemical stressors, such as childhood trauma and family dysfunction, further compound the risk of ADHD, particularly in individuals with specific genetic profiles [83,84,85].

**Figure 2:**
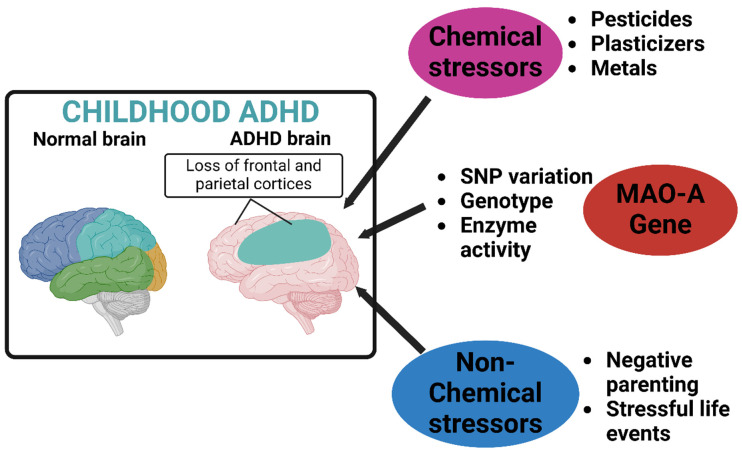
Neurodevelopmental differences in the brain of a child with ADHD compared to its typical developing peer. This figure was created using Biorender, referencing [33].

The gene–environment interaction hypothesis posits that genetic susceptibility may moderate the impact of environmental factors on ADHD’s onset and progression (Figure 2) [86]. Individuals with certain genetic variants may be more sensitive to environmental influences, such as prenatal substance exposure, leading to a higher likelihood of developing ADHD. This complex interplay between genetics and environment underscores the need for integrative approaches to prevention and intervention strategies.

## 4. Current Treatment Landscape

**Figure 3:**
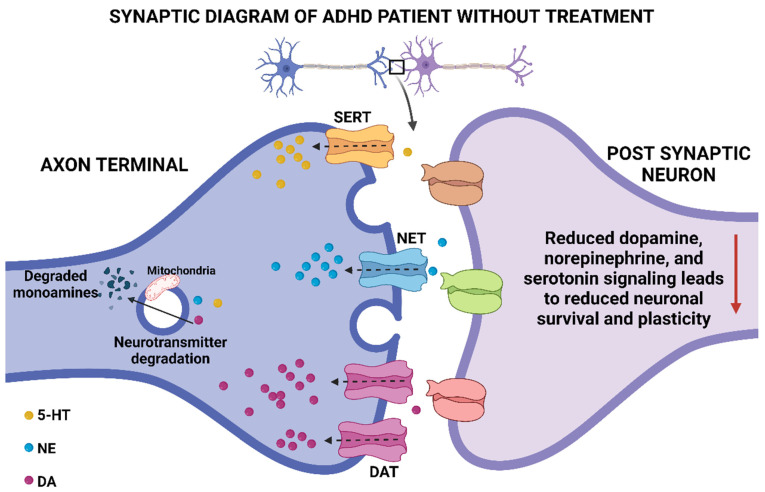
Illustration of synaptic terminal activity in ADHD patients before treatment, showing reduced dopamine and norepinephrine signaling. This figure was created using Biorender, referencing [33].

**Figure 4:**
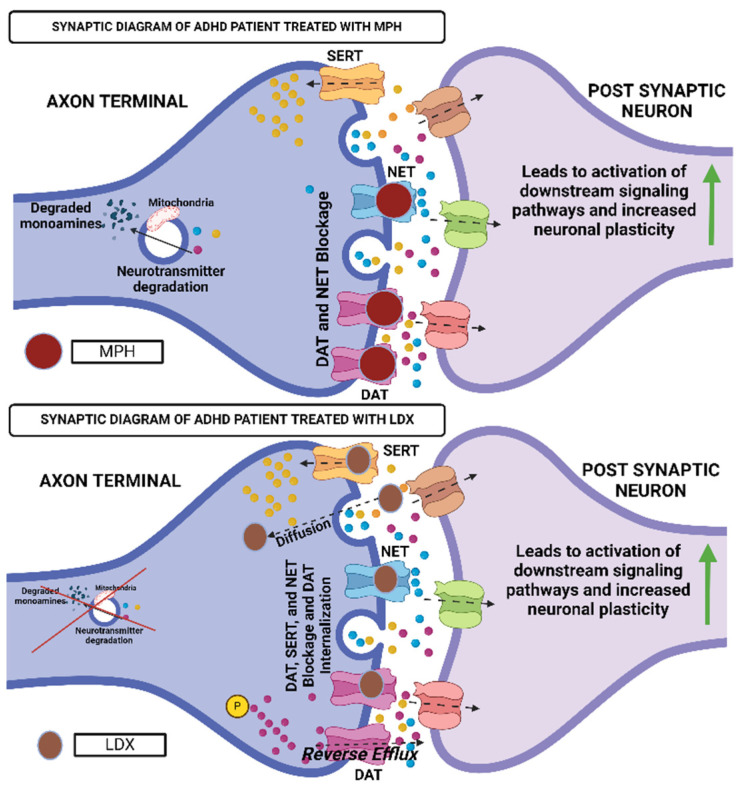
Enhanced synaptic terminal function in ADHD patients following methylphenidate and lisdexamfetamine treatment, demonstrating increased neurotransmitter availability. This figure was created using Biorender, referencing [33].

Stimulants like methylphenidate (MPH) and lisdexamfetamine (LDX) are first-line treatments for both children and adults and are particularly effective for managing inattention and executive function deficits (Figure 3 and Figure 4) [87,88,89,90,91,92,93]. MPH, a norepinephrine and dopamine reuptake inhibitor, is available in extended-release formulations like Concerta^®^ and Medikinet^®^ [33,94,95,96,97,98,99,100,101]. LDX, a prodrug converted to d-amphetamine, is often prescribed when MPH is ineffective or poorly tolerated [102,103,104,105,106,107].

Non-stimulant medications, such as atomoxetine, guanfacine, and clonidine, are used for patients with coexisting conditions like anxiety or tics, or when stimulants are unsuitable (Table 1) [108,109]. Atomoxetine, a norepinephrine reuptake inhibitor, is less prone to misuse, while guanfacine and clonidine, alpha agonists, manage aggression and sleep issues [110,111,112,113].

Treatment choices depend on individual symptom profiles, comorbidities, prior response to medications, and patient preferences. Non-stimulants are also favored in cases of substance misuse or concerns about stimulant addiction.

## 5. Current Challenges in Addressing ADHD Etiology and Treatment Gaps

Despite significant advances in ADHD treatment options, several challenges persist in providing optimal care. Access to healthcare providers remains limited in many regions, particularly in rural populations. Additionally, stigma surrounding ADHD diagnosis and treatment continues to prevent individuals from seeking help. The cost of medications, therapy, and ongoing care can be prohibitive for many families. Furthermore, there is a notable gap in evidence-based treatments tailored to adults with ADHD, as most research has historically focused on pediatric populations. Treatment adherence remains problematic, particularly among adolescents and young adults who may resist medication or struggle with consistent follow-up. Additionally, comorbid conditions such as anxiety, depression, or substance use disorders often complicate treatment planning and require integrated care approaches that may not be readily available in all healthcare settings [38].

### 5.1. Long-Term Management of ADHD

The long-term management of ADHD is fraught with complexities, particularly due to the individualized nature of treatment and the evolving needs of patients over time. While pharmacological and behavioral interventions are widely used, their efficacy and adherence vary, requiring a critical examination of current approaches. It is essential to adopt a more holistic perspective that considers the unique challenges faced by specific populations, such as adults with ADHD, females, and those in underserved communities. This section will explore these challenges and propose potential solutions, including tailored interventions, integrated care models, and emerging research on objective biomarkers for diagnosis and management.

Access to Care: Access to healthcare providers remains limited in many regions, particularly in rural and low-income populations. Telemedicine and mobile health services could be vital in enhancing access to healthcare providers, especially in underserved and remote areas. Additionally, governments and organizations are increasingly offering subsidies and affordable ADHD care options, including medications and therapy, to address the economic barriers many families face [37]. These efforts can play a significant role in improving access and ensuring equitable healthcare distribution.

Stigma Reduction: Public health campaigns, educational programs, and media platforms have crucial roles in reducing stigma. By raising awareness and promoting ADHD as a neurodevelopmental disorder rather than a behavioral issue, stigma can be minimized [37]. Schools, workplaces, and community settings must actively promote ADHD awareness to foster a more supportive environment for individuals affected by the condition. Normalizing discussions around ADHD diagnosis and treatment will encourage more individuals to seek help without fear of judgment.

Barriers in Adult ADHD Treatment: ADHD in adults remains underdiagnosed, with treatment strategies primarily focusing on pediatric populations. There is a growing need for evidence-based treatment models tailored specifically for adults. Future research should prioritize the development of adult-specific diagnostic criteria and treatment options. Additionally, emerging models, such as telepsychiatry, digital cognitive behavioral therapy (CBT), and workplace accommodations for ADHD [32], could help bridge this treatment gap and offer adults more effective management solutions.

Treatment Adherence: Improving treatment adherence is a critical challenge, particularly among adolescents and young adults who may resist medications or have difficulties with consistent follow-ups. Digital tools, including medication reminder apps and telehealth check-ins, can provide patients with timely reminders and enable better communication with healthcare providers. Furthermore, psychoeducation is essential in empowering patients and families to understand ADHD management and the importance of adhering to prescribed treatments. Behavioral interventions such as CBT can also enhance adherence by fostering engagement in self-care [32].

Collaborative Care Models for Comorbid Conditions: Given the high prevalence of comorbid conditions such as anxiety, depression, and substance use disorders among ADHD patients, collaborative care models are necessary to provide integrated care. Interdisciplinary teams of psychologists, psychiatrists, and educators can streamline diagnostic accuracy and treatment planning, leading to better outcomes [114]. Such integrated care models have been shown to significantly improve the management of ADHD and co-occurring conditions by providing tailored treatment plans that consider the patient’s overall mental health.

### 5.2. Gaps in Current Research

Despite significant progress in understanding ADHD’s mechanisms and treatment approaches, many critical research gaps remain. A comprehensive understanding of the complex interplay between genetic, neurobiological, and environmental factors contributing to ADHD’s development and persistence is still lacking [115,116,117,118,119]. There is also a pressing need to develop more effective, targeted treatments and optimize existing interventions to improve long-term outcomes for individuals with ADHD, especially in adult populations where non-pharmacological interventions are understudied [120,121]. Additionally, ADHD’s neurobiological mechanisms across different age groups and the long-term effects of current treatments are poorly understood [119]. Gender-specific manifestations and treatment responses, particularly in underdiagnosed females, are insufficiently explored.

Moreover, research into the specific epigenetic and genetic factors involved in ADHD remains underdeveloped. While several candidate genes and genetic variants have been identified, the polygenic nature of the disorder suggests a complex interaction of multiple genetic and environmental factors. The role of rare genetic variants, such as copy number variants, in ADHD pathogenesis needs further elucidation. There is also a growing need for studies on epigenetic modifications and their potential impact on ADHD symptom development, offering insights into how environmental factors might influence gene expression. Novel therapeutic approaches, including digital interventions and personalized medicine strategies, hold promise but require further validation [122]. Finally, understanding the developmental trajectory of ADHD across a lifespan, particularly how symptoms evolve during key life transitions, and clarifying the molecular signaling pathways affected by the disorder remain crucial areas for future research.

### 5.3. Inherent Subjectivity of the Diagnostic Criteria

The lack of standardized diagnostic tests has led to inconsistencies in ADHD diagnosis across different settings. As research advances, the development of more robust and objective diagnostic tools will help mitigate these challenges. Current efforts focus on neuroimaging and genetic biomarkers to provide a biological basis for diagnosis [121]. These tools have the potential to complement behavioral assessments, offering a more comprehensive and consistent diagnostic framework, particularly in borderline cases [123,124]. For example, neuroimaging could be used to detect patterns of brain activity associated with ADHD, leading to a more accurate and individualized diagnosis.

### 5.4. Sociocultural Factors

Cultural and socioeconomic factors significantly impact the diagnosis and treatment of ADHD. Minority populations and individuals from low-income backgrounds are more likely to be underdiagnosed due to cultural biases, language barriers, and limited access to healthcare. To address these disparities, healthcare professionals must be trained in culturally sensitive diagnostic practices. Culturally adapted screening tools, community health workers, and school-based interventions could help bridge the diagnostic gap and ensure that more individuals receive timely ADHD diagnosis and care. Additionally, language-appropriate educational materials and support services are crucial to improving awareness and understanding of ADHD in diverse communities.

### 5.5. Comorbidity

Another significant challenge is the comorbidity of ADHD with other neurodevelopmental or mental health disorders. The presence of comorbid conditions alongside ADHD significantly complicates both diagnosis and treatment, often resulting in misdiagnosis or delayed intervention. Many individuals with ADHD experience concurrent mental health conditions, such as anxiety disorders, depression, or learning disabilities. These overlapping conditions can mask or exacerbate ADHD symptoms, making it challenging to determine the primary source of difficulties. Research indicates that up to 60% of individuals with ADHD have at least one co-occurring condition, necessitating careful consideration in treatment planning. These comorbidities can also lead to a more complex clinical presentation, requiring a more comprehensive assessment and tailored treatment approaches [125,126,127]. A comprehensive approach to addressing comorbidity in ADHD requires careful assessment and individualized treatment planning. The development of more robust and objective diagnostic tools, including neuroimaging and genetic markers, could enhance the accuracy and reliability of ADHD identification. Improved coordination among healthcare professionals, such as pediatricians, psychologists, and psychiatrists, can also help streamline the diagnostic process and ensure a more comprehensive assessment. Healthcare providers should conduct thorough evaluations that screen for multiple conditions simultaneously. Treatment strategies often need to be integrated, addressing both ADHD and co-occurring conditions. This may involve combining medication management with targeted behavioral interventions, cognitive behavioral therapy, and educational support.

### 5.6. Gender-Specific Diagnostic Criteria and Treatment Strategies

Females with ADHD often present with different symptoms, such as inattentiveness and internalizing behaviors, which may be less noticeable than the hyperactive–impulsive symptoms traditionally associated with ADHD. This contributes to underdiagnosis and delayed treatment in females. Expanding research into gender-specific diagnostic criteria, including symptoms more prevalent in females, is critical. Moreover, treatment strategies tailored to the unique experiences of females with ADHD, particularly those that address emotional dysregulation and executive function challenges, could significantly improve outcomes.

## 6. Conclusions

ADHD remains a complex condition with substantial implications for individuals, families, and society. Advances in understanding its neurobiology and treatment options have improved management strategies, yet significant challenges persist, particularly in addressing diagnostic subjectivity, sociocultural disparities, and comorbid conditions. Future research should prioritize longitudinal studies, innovative therapeutic approaches, and targeted interventions to bridge existing gaps. Enhanced public awareness and equitable healthcare access are crucial in improving the quality of life for those affected by ADHD. This review underscores the importance of a multidisciplinary, patient-centered approach to navigate the complexities of ADHD effectively.

## Figures and Tables

**Table 1:** Similarities and differences between stimulant and non-stimulant medications.

Features	Stimulant Medications	Non-Stimulant Medications
Common Medications	Methylphenidate (Ritalin, Concerta), Amphetamine (Adderall, Vyvanse)	Atomoxetine (Strattera), Guanfacine (Intuniv), Clonidine (Kapvay)
Mechanism of Action	Increases dopamine and norepinephrine activity in the brain by blocking their reuptake at synapses	Regulates norepinephrine levels or affects adrenergic receptors, without directly boosting dopamine levels
Onset of Action	Fast-acting (within 30–60 min)	Slower onset (can take 2–6 weeks for full effect)
Window of Effectiveness	Shorter half-life, often requiring multiple doses per day or extended-release forms	Longer half-life, typically taken once a day
Effectiveness	Highly effective in ~70–80% of patients	Effective in ~50–70% of patients, often considered secondary when stimulants fail
Side Effects	Insomnia, appetite suppression, increased heart rate, anxiety, tics	Fatigue, drowsiness, dry mouth, irritability, gastrointestinal issues
Risk of Addiction	Higher risk, controlled substances with potential for misuse and dependence	Low to no risk of addiction or dependence
Use in Pregnancy and Breastfeeding	Generally not recommended due to potential risks to the fetus and infant	Caution required; atomoxetine may be safer than stimulants
Drug Interactions	Can interact with antidepressants, antacids, and medications for high blood pressure or hyperthyroidism	Fewer drug interactions, but can exacerbate conditions like fatigue or low blood pressure
Best Suited For	Patients who need rapid symptom control and have no contraindications for stimulant use	Patients who cannot tolerate stimulant side effects, or have coexisting conditions like tics or anxiety
Who Should Avoid	People with a history of substance abuse, heart conditions, or certain psychiatric disorders	Those with severe hypertension, or conditions aggravated by fatigue or low blood pressure
